# Experiences of Habitual Physical Activity in Maintaining Roles and Functioning among Older Adults: A Qualitative Study

**DOI:** 10.1155/2016/1459597

**Published:** 2016-12-18

**Authors:** Hadeel Halaweh, Ulla Svantesson, Carin Willén

**Affiliations:** ^1^Department of Physiotherapy, Institute of Neuroscience and Physiology, Sahlgrenska Academy, University of Gothenburg, Gothenburg, Sweden; ^2^Department of Physiotherapy & Rehabilitation, Faculty of Health Professions, Al-Quds University, Jerusalem, State of Palestine

## Abstract

Physically active older adults have reduced risk of functional restrictions and role limitations. Several aspects may interrelate and influence habitual physical activity (PA). However, older adults' own perspectives towards their PA need to be addressed. The aim of this study was to explore the experiences of habitual physical activity in maintaining roles and functioning among older adult Palestinians ≥60 years. Data were collected through in-depth interviews based on a narrative approach. Seventeen participants were recruited (aged 64–84 years). Data were analyzed using a narrative interpretative method.* Findings*. Three central narratives were identified,* “keep moving, stay healthy,”* “*social connectedness, a motive to stay active,”* and “*adapting strategies to age-related changes.” Conclusion*. Habitual physical activity was perceived as an important factor to maintain functioning and to preserve active roles in older adults. Walking was the most prominent pattern of physical activity and it was viewed as a vital tool to maintain functioning among the older adults. Social connectedness was considered as a contributing factor to the status of staying active. To adapt the process of age-related changes in a context to stay active, the participants have used different adapting strategies, including protective strategy, awareness of own capabilities, and modifying or adopting new roles.

## 1. Introduction

For individuals to contribute to society, good health is a key, and an active life enhances better chance of being healthy [1, 2]. Participation in physical activity (PA) has an important role in improving health, including improved treatment of many diseases [3–7]. The evidence of health benefits of PA is stronger for adults above 65 years old than any other age group, since the consequences related to inactivity are more severe in this age group [8]. PA can help older adults prevent a decline in health related quality of life (HRQoL) and improve their enjoyment of life [9]. In older adults, habitual physical activity includes leisure time, transportation (e.g., walking or cycling), household chores, sports or planned exercise, and family and community activities [8]. Patterns of PA may change with ageing [10] and may differ between different populations. In the West Bank (Palestine), the prevalent domain of PA among older adults revolves around activities such as walking, gardening, and household chores mainly among women [11, 12].

Evidence suggests that active older adults may have better physical functioning including balance, muscle strength, coordination, and endurance [13–17]. This interaction is positively reflecting on quality of life and feelings of well-being in older adults [14, 15]. Consequently, good physical functioning enables the older adults to perform more integrated functional tasks which include activities of daily living and the fulfillment of social roles as well as recreational activities [13, 18]. With ageing, components of physical functioning are influenced by age-related changes occurring in the body composition [18–21]. These physical changes may restrict functional abilities and may lead to role limitations in older adults [20, 22, 23]. For adapting age-related changes, older adults may need to develop new strategies that require actions and lifestyle' modifications, in order to enhance functioning and maintaining own roles [24].

Active participation by maintaining own roles (i.e., employee, spouse, parent, volunteer, grandparent, and caregiver) is related to maintenance of health, functioning, and well-being among older adults [25–30]. Participation is defined as “involvement in a life situation,” meaning being included or engaged in an area of life, in addition to the subjective aspects of these life areas such as satisfaction, fulfillment, and enjoyment. The individual's degree of involvement is influenced by the individual's level of functioning and society's response, an interaction that may facilitate or inhibit participation [31]. Therefore, societal involvement could be understood in the light of maintaining own roles [32]. Older adults who are involved in a variety of activities and occupying active roles are more likely to age with a sense of satisfaction and higher self-efficacy [29, 33].

The present qualitative study is a part of a larger project, studying factors of active ageing including physical activity [12] and physical functioning [34] in older adults. These quantitative studies have provided evidence of the correlates for PA and physical functioning among the older adults in the West Bank (Palestine). However, older adults' own perspective towards their physical activity and functioning is not explored yet. Qualitative studies can provide better insight into older adults' experiences that cannot be elicited through quantitative studies. Thus, the aim of this study was to explore the experiences of habitual physical activity in maintaining roles and functioning in community dwelling older adult Palestinians who were 60 years old and older at the time of the data collection.

## 2. Methods

A narrative approach was applied as a qualitative research design [35, 36]. The method was chosen because narrative descriptions can enhance knowledge and understanding of the complexity of older adults' experiences of habitual physical activity related to maintaining own roles and functioning.

The sample was selected from a previous related cross sectional study [34], addressing physical functioning and fall-related efficacy among older adults (*n* = 176). Recruitment procedure was arranged through coordination with different community and physiotherapy centres in the West Bank (Palestine). The inclusion criteria were being older adults (aged ≥60 years), living in the West Bank (Palestine), being able to walk with or without walking aids, living at own homes within a family or alone, and having no communication deficits that would make interviewing impossible.

A total of seventeen participants were recruited in this study, age ranging between 64 and 84 years. All participants were fully independent in performing their basic activities of daily living (BADL) [37], meaning they were able to perform basic activities of daily living including dressing, toileting, and feeding without assistance from another person, and four of the participants were partially independent in bathing. Participants' demographic and clinical characteristics are illustrated in Table 1.

All participants were given verbal and written information about the aim of the study and they signed an informed consent form. The participants were ensured confidentiality and informed that their participation was voluntary and that they could drop out of the study at any time. The study received ethical approval from the Research Ethics Committee of Al-Quds University, Palestine (Ref number 1/REC/13), which complies with the Declaration of Helsinki.

### 2.1. Data Collection

Data were collected using in-depth individual interviews that involves conducting intensive individual interviews with the participants [38]. As mentioned above this study is a part of a larger project studying physical functioning and physical activity; interviewing was thus based on two to three interviews with the participants. All interviews were conducted face to face by the first author. The participants were informed that the interview would be open and focused on their own experiences of habitual physical activity. The interviews were conducted using a narrative approach [35, 36]. In this approach, informants use their spontaneous language in the narration of different life events connected to their experiences; the interviews were designed to elicit narratives, a strategy that is based on a narrative inquiry approach. And some predetermined broad questions guided the interviews:-

- Can you please tell me about your daily life activities including household chores, walking, and gardening?
- How do you experience your physical activity and fitness at this stage? How do you see that may influence your functioning and roles?
-  How do you think you can maintain or adapt your physical activity habits? In addition, all participants were asked to describe their current self-rated fitness: how you evaluate your physical fitness?

All interviews took place in the natural environment at the participants' homes, which helped to enrich the narrative material by having realistic images of how things relate and communicate in their natural surroundings. During the interviews the researcher also had the possibility to make participants' observations [39], by joining the participants and having conversations with them while performing their activities at their homes, work, or garden. That gave access to richer information about how the participants' live their daily routine. Field notes were recorded after each interview.

Data were collected between April 2013 and summer 2014. Each interview lasted 60 to 120 minutes; the interviews were digitally audio-recorded. In addition, with permission from the interviewees, some interviews were video-recorded. All interviews were transcribed verbatim by the first author and translated From Arabic into English by the first author in collaboration with a bilingual translator.

### 2.2. Data Analysis

The analyzing process was conducted using the interpretative narrative method [35, 36]. The software NVivo [40] was used as a helpful tool for analysis. To obtain a sense of the wholeness of the material, the transcribed data were read several times and were discussed in depth by the authors through dynamic dialogues. Also, the video analysis of the interviews contributed to richer data material. Events and happenings that were central to the study's aim became apparent; accordingly, significant events from the participants' narratives were identified. A significant event is defined as a central block in the structuring of the narrative [41]. Data from all interviews were organized by synthesizing the data rather than by separating the data into constituent parts [35]. An initial draft of evolving plots was formulated, and the interpretative analysis was carried out by building up a plot within the notion of the hermeneutic circle where the development of the text was revealed by the back and forth movements from parts to whole [35, 42]. Data were reinterpreted in discussions between the authors, exchanges that helped create an interpretative space for testing and working with the findings until consensus was reached.

## 3. Results

**Figure 1:**
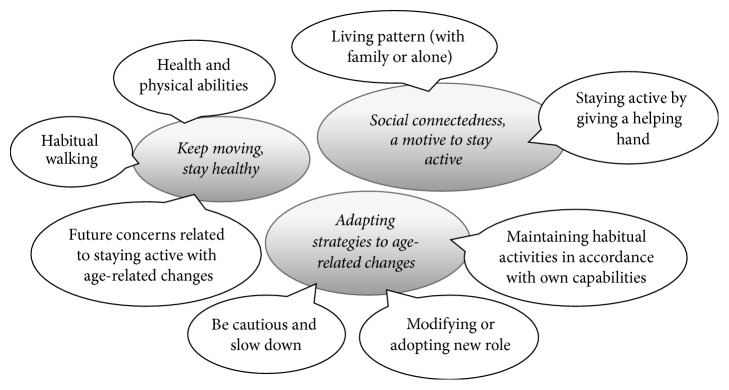
Central narratives and related significant events.

Three central narratives were identified, the first narrative was* “keep moving, stay healthy, ****”*** the second narrative was “*social connectedness, a motive to stay active, ****”*** and the third narrative was “*adapting strategies to age-related changes. ****”*** In-depth descriptions of all narratives and related significant events are presented below and are illustrated in Figure 1.

*(1) Keep Moving, Stay Healthy*. Health and physical abilities were considered major factors that influence functioning and maintaining roles of older adults in the community. The participants' experiences of staying active were connected to positive state of well-being. Therefore, in our analysis, we have considered “health and physical abilities” as the first significant event of this narration.

I am taking care of my health, because if my health is good, my fitness will be good. This is very important to stay active and to maintain my role in the community. I'm trying to maintain my activity as much as I can. I take short walks, especially in nice weather, and in a reasonable limit, because anything when you exceed its limit, it definitely will turn against it. (Participant 10)

Through this narration, optimal health according to the participants' own perspectives was connected with performing daily activities. The pattern of physical activity was centered on activities such as walking, gardening, and housework.

I keep moving, better than sitting and becoming lazy. Every morning, I get up and I walk among the trees. Walking is good, even it eases the body, I don't like to sit for a long period, always I move my legs, otherwise my legs will be tightened. (Participant 9)

The second significant event in this finding was related to “habitual walking” as a prominent pattern of PA among the studied population. An expression that was commonly used by almost all the participants in the interviews was “passing away with my own dusty shoes,” indicating their wish to stay healthy and able to walk until the last day of their life. The participants talked about the importance of walking and its positive effect on physical functioning.

I keep moving at home, and I walk fast. I get tired sometimes when I walk up hills and going upstairs. Still, I keep moving, otherwise I'll be incapicitated. (Participant 12)

Through this finding, the state of being active and healthy was connected to concern about potential future limitations. Many participants expressed their concerns about the future, which were mostly related to the obsession of staying healthy and active in a context of their fear of becoming a burden to others. Here, the third significant event became apparent as “future concerns related to staying active.” The participants connected their ability to move and to function well to their state of being independent and maintaining owns roles.

I walk every day, sometimes it depends on the weather, if it's too hot, I prefer to take evening walks. Walking helps me keep going in this life; I want to stay active and healthy. I don't like to bother nobody, or to be a burden to anybody. (Participant 14)

Most of the participants reflected on how physical decline may hamper their physical activity and consequently may lead to limited functioning and to restricted active role. In this context, the participants' experiences were displayed along with their perception of age-related changes.

You lose something as you are ageing, I've retreated a little but I don't complain. I feel that I get older, my body needs more attention, it is not as strong as it used to be before. I always say to my neighbor who always complains, my chest ache, my back ache. I tell her don't complain just move, go out, don't be home bounded. (Participant 6)

The scene in this finding appeared more knotty, in certain circumstances where age-related changes were associated with tough events such as fractures or diseases. That was expressed by two participants (3, 5).

I was active. I was taking care of myself until a year ago, when I fell here in front of the door. I was trying to push the key, when I fell and I hit my head and broke my hip. They took me to the hospital. I underwent surgery for my broken hip. My health is up and down; all what I need is to be able to serve myself. My relatives help me; I am not able to cook or to wash. Before, I was doing everything by myself, but nowadays, I can't. I became tired and I walk with a walker. (Participant 5)

*(2) Social Connectedness, a Motive to Stay Active*. In the second narrative, social connectedness contributed to the status of staying active, functioning, and maintaining own role. Our findings showed that living pattern (living with family or alone) had a great influence on how the participants reflected on their experiences. Hence, “living pattern” was considered as a significant event of this narration. Most of the participants related their status of being physically active with their capabilities of maintaining their roles at both familial and community levels. There was a combination of physical functioning while doing their activities with a sense of connectivity with their surroundings.

We have so sweet grandsons and daughters. As you see, children all over the house. This makes me happy. When they go, the house is empty. I start to call them come Teta (Yousef, Zeinah), I am preparing the breakfast. All of them love my food. And every day I have visitors, my neighbors come over for coffee, thanks god my house always open for people. I like having social life that helps me staying active and happy. We always have something to do. (Participant 6)

The influence of the interaction of living pattern and the state of being active and functioning was more prominent among elderly women who lived alone, as their living experiences were entirely altered when they became widows or living alone. They connected the state of being lonely with more limited roles.

My role differs to a large extent, the house was fulI, now I am living alone, but I do everything, I cook, wash my clothes, and I clean the house. I am not active as before, I just go out for visiting my relatives, they live nearby, but I don't have strength like before. (Participant 8)

To a large extent, the state of connectedness among the participants was situated by their familial and social links. However, there was also a scene where some participants could manage to create their own connectedness and situate themselves as persons who are still active and capable of helping others (people, pets, and plants). The participants' highlighted the vitality of this kind of connectedness based on their perceptions that they are still active and being needed by others. In view of that, the second significant event in this narration was underlined as “staying active by giving a helping hand.” Participant 11 who is a healer (treating different pathological conditions using traditional natural modalities) says

People come to my house from everywhere. I treat everyone, old and young, men and women, those complain of neck pain or back patch. I fix cracked legs and hands. All these people appreciate me. Sometimes I have like four or five visits per day. That helps me keep going and stay active. (Participant 11)

Some participants viewed this connectedness by being capable of helping others as a mutual positive link, which motivated them to stay active and functioning.

I live by myself, but I have many trees. They need me. I am taking care of them, the snow destroyed everything, and most of the trees gone away, also a fig tree, very delicious one is also gone. That saddened me a lot. Still, I keep working around the house. I mean, every day an hour or more, I dig around the olive trees. That helps me to stay active by doing something good. (Participant 12)

*(3) Adapting Strategies to Age-Related Changes*. The participants described their current pattern of physical activity and its influence on maintaining functioning and active roles, by developing new strategies to adapt the process of age-related changes. This was transformed into actions which helped them to formulate a realistic image of their experiences. The participants in this narrative talked about three different strategies. In our analysis, we have considered these strategies as significant events of this narrative. The first strategy was* “be cautious and slow down.” *The second strategy was “*maintaining habitual activities in accordance with own current capability.”* And the third strategy was “*modifying or adopting new role.” *

According to the majority of the participants, the first strategy “*be cautious and slow down”* was connected to their perceptions of age-related changes; they were aware that their bodies are not responsive as fast as before. Accordingly, they may need to slow down; some participants described this strategy as a protective strategy, as they tended to modify their choice of movements by readjusting their walking rhythm, so their steps get slower and more thoughtful to prevent falling. This adapting strategy was used by most of the participants to feel safer and to be able to carry out their daily activity routine.

I pay attention; I need to figure out where to put my feet. My daughter lives in a mountainous area, where the road to her house is bumpy. When I go to visit her, she tells me to grab her hand; I tell her, just you go. I walk on my comfort, I am cautious and I watch my steps. (Participant 6)

Some participants described how they made modifications in their daily activities style, in order to be able to stay active; solutions included starting to use a walking aid (cane) when going out for a walk or for a social event.

Sometimes when I go to visit my sister, there is a sharp downhill, so I take a cane. It helps me going down hills and I feel safer. (Participant 7)

Another solution was declared by some participants (mainly women) that they preferred to be accompanied by others when going out; that gave them a sense of security from their own perspective.

I was walking and visiting my relatives, but after the surgery, I can't walk well, just I walk a little, I need to be accompanied when I go out, I don't feel safe to go alone, I am afraid of falling again. (Participant 5)

The second adapted strategy to age-related decline was “*maintaining habitual activities in accordance with own current capability.” *Most participants perceived themselves as active persons within their current physical potentials; they illustrated to which extent they were capable of carrying out these activities and maintaining their roles.

I am doing what I am supposed to do. I am taking care of my family and house within my abilities. The most important thing is not to surrender life, giving up and saying I am old, or sitting and staying at home. No, I go out, I move. If I become old, shall I sit and wait for my death? Death will come anyhow, but one needs to live, to move. (Participant 8)

Maintaining habitual activities in accordance with own current capability was also reflected on adapting current leisure activities that some of the participants were used to do. Most of the participants talked about how important it is for them to find their own pace while doing activities.

Look at this [her traditional dress]. I've embroidered it, but now I am doing simple things. I was doing harder work before, but now with ageing, I can manage to do only simpler things. I am happy that I am still capable to do something. (Participant 13)

A few participants found the third adapting strategy “*modifying or adopting new role” *as a helpful tool for them to adapt age-related changes. That might be grounded upon their perspective of taken on changes within their bodies and to which extent they were capable of carrying out these changes to be able to stay active and to maintain own role.

I keep myself busy, after retirement, I've made a small business, and I am a peasant in nature, we have land for cultivation, if I have free time, I just fill it continuously. That helps me stay active at this stage of life. (Participant 17)

## 4. Discussion

### 4.1. Methodological Considerations

Relevant studies have indicated that physically active older adults have a better chance to perform more integrated functional tasks which include activities of daily living and the fulfillment of social roles as well as recreational activities [13, 18]. The evidence of these studies is mostly derived from quantitative research. In the present study, we wanted to explore the experiences of older adults related to their physical activity in maintaining their functioning and own roles. Therefore, a qualitative narrative approach was used as a form of qualitative research in which participants shape and give voice to their experiences [43, 44]. The capability of narrative to combine diverse events, happenings, and actions of human experiences made it merited to be applied for exploring the experiences of older adults in the present study. These experiences were better comprehended by focusing on the human actions within the dynamics of the surrounding environment.

Conducting the interviews at the participants' natural environment (homes) gave the researcher an access to their actual situations, which helped enrich the material and provide an additional valuable dimension of the derived data for the analysis. The relationship between the interviewer and the interviewees has been developed progressively through several interviews. So information derived at different times contributed to the consistency of the derived information from the interviews. At the first interview, the researcher was positioned as a researching physiotherapist testing physical functioning. At a later interview, the interviewees were invited to participate in the current study. Recurrent interviews with the participants gave a chance to create a comfortable interviewing atmosphere, which helped the researcher build a trusting connection with the participants and encouraged the participants to talk more freely.

As most qualitative studies, the goal of this study is not to generalize the analysis but rather to provide a rich, contextualized understanding of some aspect of human experience through the intensive study of particular cases [45]. The used interviewing guide in this study was based on a general rule on sample size for in depth interviews, indicating when the same stories, themes, and topic are emerging from the interviewees; then a sufficient sample size has been reached [46].

### 4.2. Reflection on the Findings

Older adults who are physically active have reduced risk of functional restrictions and role limitations [8]. Corresponding with our findings in the first narrative “keep moving, stay healthy,” the participants connected their status of being healthy and active with the nature of their habitual activity. Walking was the most prominent pattern of physical activity; almost all participants related their state of well-being and functioning to their ability to walk. This finding can be understandable as walking is viewed as one of the most recommended and popular forms of PA among older adults and it can easily be adapted into daily lifestyle [47].

Through this finding, the participants have expressed how being active was considered of great importance for them. The reflection of the participants' experiences was related to a synthesis of what people do in relation to who they are and to who they want to become [48]. They were obsessed with staying healthy and active in a context of their fear of becoming a burden to others in the future. Most of the participants connected their abilities to perform their daily activities without assistance from others to their physical activity status. Staying active from the participants' perspective was a very important key factor that helped them to maintain their roles at both individual and familial levels. Findings support the activity perspective and the role enhancement among older adults within the view of active ageing [49].

In the second narration “social connectedness, a motive to stay active” the significant event “living pattern (with family or alone)” was especially imperative when looking at older women's lives. Active ageing and well-being among older adults in Palestine are reflected in family interaction and social roles within the families. Recently there has been a shift from extended families where older adults are surrounded by their children and grandchildren to nuclear family patterns where older adults live alone, especially older women who are more likely to spend the later stage of their lives alone [11]. This transition influences quality of life as well as physical health, as older women reported better physical health when they were living with family members compared to women living alone [50]. Living alone and low social participation were found to be significant risk factors for later disability onset [51]. Older adults who live alone report more fatigue and more health difficulties than older adults who do not live lonely [26, 52].

Inspired by the third narration, it seems that adapting to age-related changes is a process that requires individual skills, analogous to older adults' beliefs about their health and ability to control the decline of the ageing body, which may contribute to the actual loss of function later on in the future life [53]. A process consists of both internal and external dimensions; the internal dimension locates within the older adults themselves represented by their awareness of own potentials and capabilities, to come across appropriate solutions that help them adapt age-related changes in order to stay active, functioning, and maintaining own role. This dimension was embodied in strategies such as slowing down and being more cautious or using an assistive device (cane) while going out for a walk.

The external dimension was located in the participants' surroundings including people and settings. The external dimension was described by the WHO [31], as environmental factors that include physical, social, and attitudinal environment in which people live; these factors can have a positive or negative influence on the individual's capacity to adapt and may contribute to enhance capabilities in adapting the age-related changes. Some participants described social support from others as a strategy to help overcome boundaries that prevent them from staying active.

## 5. Conclusion

The participants in this study perceived habitual physical activity as an important factor to maintain functioning and to preserve active roles. Walking was the most prominent pattern of physical activity among the older adults in this study, and it was viewed as a vital tool to maintain physical activity and functioning. Social connectedness was considered by most of the participants as a contributing factor to the status of staying active as well as maintaining own role. To adapt the process of age-related changes and its impact on maintaining functioning and active roles, the participants used different adapting strategies, including protective strategy to prevent falling, awareness of own capabilities, and modifying or adopting new roles.

## Figures and Tables

**Table 1:** Participants' demographic and clinical characteristics.

Characteristics	*N* = (17)	Participant #	Age (years)	Gender	Marital status
Age (years), mean (SD)	72.7 (8.35)	1	77	Female	Widow
Living status, *n* (%)		2	80	Female	Widow
With family, alone	10 (59), 7 (41)	3	67	Male	Married
Diagnosed disease, *n* (%)	15 (88)	4	64	Male	Married
Hypertension	7 (41)	5	83	Female	Widow
Diabetes	9 (53)	6	64	Female	Married
Cardiovascular	3 (18)	7	84	Male	Widower
Musculoskeletal	10 (59)	8	67	Female	Widow
Other chronic diseases	2 (12)	9	65	Female	Married
Assistive devices, *n* (%)	11 (65)	10	65	Male	Married
Glasses	10 (60)	11	83	Female	Widow
Walking aid (cane)	4 (24)	12	64	Female	Single
Self-rated fitness, *n* (%)		13	78	Female	Married
Poor	5 (29)	14	83	Male	Married
Quiet good	7 (41)	15	67	Female	Married
Good	3 (18)	16	81	Male	Married
Very good	2 (12)	17	65	Male	Married

## References

[1] Kalache A., Gatti A. (2003). Active ageing: a policy framework. *Advances in Gerontology*.

[2] Akune T., Muraki S., Oka H. (2014). Exercise habits during middle age are associated with lower prevalence of sarcopenia: the ROAD study. *Osteoporosis International*.

[3] Beitz R., Dören M. (2004). Physical activity and postmenopausal health. *Journal of the British Menopause Society*.

[4] Brach J. S., Simonsick E. M., Kritchevsky S., Yaffe K., Newman A. B. (2004). The association between physical function and lifestyle activity and exercise in the health, aging and body composition study. *Journal of the American Geriatrics Society*.

[5] Pedersen B. K., Saltin B. (2006). Evidence for prescribing exercise as therapy in chronic disease. *Scandinavian Journal of Medicine and Science in Sports*.

[6] Ferencz B., Laukka E. J., Welmer A.-K. (2014). The benefits of staying active in old age: physical activity counteracts the negative influence of PICALM, BIN1, and CLU risk alleles on episodic memory functioning. *Psychology and Aging*.

[7] Fox K. R., Ku P.-W., Hillsdon M. (2015). Objectively assessed physical activity and lower limb function and prospective associations with mortality and newly diagnosed disease in UK older adults: an OPAL four-year follow-up study. *Age and Ageing*.

[8] World Health Organization Global recommendations on physical activity for health. http://apps.who.int/iris/bitstream/10665/44399/1/9789241599979_eng.pdf.

[9] Choi M., Prieto-Merino D., Dale C. (2013). Effect of changes in moderate or vigorous physical activity on changes in health-related quality of life of elderly British women over seven years. *Quality of Life Research*.

[10] DiPietro L. (2001). Physical activity in aging: changes in patterns and their relationship to health and function. *The Journals of Gerontology Series A: Biological Sciences and Medical Sciences*.

[11] Palestinian Central Bureau of Statistics Dissemination and analysis of census findings: the conditions and requirements of elderly care in the Palestinian territory 1997–2007. http://www.pcbs.gov.ps/Portals/_PCBS/Downloads/book1647.pdf.

[12] Halaweh H., Willen C., Grimby-Ekman A., Svantesson U. (2015). Physical activity and health-related quality of life among community dwelling elderly. *Journal of Clinical Medicine Research*.

[13] Dipietro L. (1996). The epidemiology of physical activity and physical function in older people. *Medicine and Science in Sports and Exercise*.

[14] Fusco O., Ferrini A., Santoro M., Lo Monaco M. R., Gambassi G., Cesari M. (2012). Physical function and perceived quality of life in older persons. *Aging Clinical and Experimental Research*.

[15] Garatachea N., Molinero O., Martínez-García R., Jiménez-Jiménez R., González-Gallego J., Márquez S. (2009). Feelings of well being in elderly people: relationship to physical activity and physical function. *Archives of Gerontology and Geriatrics*.

[16] RoyChoudhury A., Dam T.-T. L., Varadhan R., Xue Q.-L., Fried L. P. (2014). Analyzing feed-forward loop relationship in aging phenotypes: physical activity and physical performance. *Mechanisms of Ageing and Development*.

[17] Halaweh H., Willén C., Svantesson U. (2016). Association between physical activity and physical functioning in community-dwelling older adults. *European Journal of Physiotherapy*.

[18] Spirduso W. W., Francis K. L., Macrae P. G. (2005). *Physical Dimensions of Aging*.

[19] St-Onge M.-P., Gallagher D. (2010). Body composition changes with aging: the cause or the result of alterations in metabolic rate and macronutrient oxidation?. *Nutrition*.

[20] Lindle R. S., Metter E. J., Lynch N. A. (1997). Age and gender comparisons of muscle strength in 654 women and men aged 20–93 yr. *Journal of Applied Physiology*.

[21] Demontis F., Piccirillo R., Goldberg A. L., Perrimon N. (2013). Mechanisms of skeletal muscle aging: insights from *Drosophila* and mammalian models. *Disease Models & Mechanisms*.

[22] Chen C.-M., Chang W.-C., Lan T.-Y. (2015). Identifying factors associated with changes in physical functioning in an older population. *Geriatrics and Gerontology International*.

[23] Goodpaster B. H., Park S. W., Harris T. B. (2006). The loss of skeletal muscle strength, mass, and quality in older adults: the Health, Aging and Body Composition Study. *The Journals of Gerontology—A Biological Sciences and Medical Sciences*.

[24] Kielhofner G. (2002). *A Model of Human Occupation: Theory and Application*.

[25] Adams K. B., Leibbrandt S., Moon H. (2010). A critical review of the literature on social and leisure activity and wellbeing in later life. *Ageing & Society*.

[26] Gilmour H. (2012). Social participation and the health and well-being of Canadian seniors. *Health Reports*.

[27] Heaven B., Brown L. J. E., White M., Errington L., Mathers J. C., Moffatt S. (2013). Supporting well-being in retirement through meaningful social roles: systematic review of intervention studies. *Milbank Quarterly*.

[28] Adelmann P. K. (1994). Multiple roles and psychological well-being in a national sample of older adults. *Journals of Gerontology*.

[29] Nordenmark M. (2004). Multiple social roles and well-being: a longitudinal test of the role stress theory and the role expansion theory. *Acta Sociologica*.

[30] Levasseur M., St-Cyr Tribble D., Desrosiers J. (2009). Meaning of quality of life for older adults: importance of human functioning components. *Archives of Gerontology and Geriatrics*.

[31] World Health Organization (2001). *ICF: International Classification of Functioning, Disability and Health*.

[32] Piškur B., Daniëls R., Jongmans M. J. (2014). Participation and social participation: are they distinct concepts?. *Clinical Rehabilitation*.

[33] Phillipson C., Baars J. (2007). Social theory and social ageing. *Ageing in Societies*.

[34] Halaweh H., Willen C., Grimby-Ekman A., Svantesson U. (2016). Physical functioning and fall-related efficacy among community-dwelling elderly people. *European Journal of Physiotherapy*.

[35] Polkinghorne D. E. (1995). Narrative configuration in qualitative analysis. *International Journal of Qualitative Studies in Education*.

[36] Riessman C. K. (2008). *Narrative Methods for the Human Sciences*.

[37] Brorsson B., Asberg K. H. (1984). Katz index of independence in ADL. Reliability and validity in short-term care. *Scandinavian Journal of Rehabilitation Medicine*.

[38] Legard R., Keegan J., Ward K. (2003). Qualitative research practice: a guide for social science students and researchers. *depth Interviews*.

[39] Alsaker S., Bongaardt R., Josephsson S. (2009). Studying narrative-in-action in women with chronic rheumatic conditions. *Qualitative Health Research*.

[40] Edhlund B. *NVivo 10 Essentials*.

[41] Mattingly C. (1998). *Healing Dramas and Clinical Plots: The Narrative Structure of Experience*.

[42] Ricoeur P. (1976). *Interpretation Theory: Discourse and the Surplus of Meaning*.

[43] Kleinman A. (1988). *The Illness Narratives: Suffering, Healing, and the Human Condition*.

[44] Sakalys J. A. (2003). Restoring the patient's voice. The therapeutics of illness narratives. *Journal of Holistic Nursing*.

[45] Polit D. F., Beck C. T. (2010). Generalization in quantitative and qualitative research: myths and strategies. *International Journal of Nursing Studies*.

[46] Boyce C., Neale P. (2006). *Conducting in-Depth Interviews: A Guide for Designing and Conducting in-Depth Interviews for Evaluation Input*.

[47] Atalay O. T., Cavlak U. (2012). The impact of unsupervised regular walking on health: a sample of Turkish middle-aged and older adults. *European Review of Aging and Physical Activity*.

[48] Nyman A., Josephsson S., Isaksson G. (2014). A narrative of agency enacted within the everyday occupations of an older Swedish woman. *Journal of Occupational Science*.

[49] World Health Organization (2002). *Active Ageing, A Policy Framework*.

[50] Imam A. (2010). *Palestinian Elderly Women's Needs and Their Physical and Mental Health*.

[51] Lund R., Nilsson C. J., Avlund K. (2010). Can the higher risk of disability onset among older people who live alone be alleviated by strong social relations? A longitudinal study of non-disabled men and women. *Age and Ageing*.

[52] Deng J., Hu J., Wu W., Dong B., Wu H. (2010). Subjective well-being, social support, and age-related functioning among the very old in China. *International Journal of Geriatric Psychiatry*.

[53] Boult C., Altmann M., Gilbertson D., Yu C., Kane R. L. (1996). Decreasing disability in the 21st century: the future effects of controlling six fatal and nonfatal conditions. *American Journal of Public Health*.

